# Coulomb-actuated microbeams revisited: experimental and numerical modal decomposition of the saddle-node bifurcation

**DOI:** 10.1038/s41378-021-00265-y

**Published:** 2021-05-28

**Authors:** Anton Melnikov, Hermann A. G. Schenk, Jorge M. Monsalve, Franziska Wall, Michael Stolz, Andreas Mrosk, Sergiu Langa, Bert Kaiser

**Affiliations:** 1 Fraunhofer Institute for Photonic Microsystems IPMS, Dresden, 01109 Germany; 2 Arioso Systems GmbH, Dresden, 01109 Germany; 3 Brandenburg University of Technology Cottbus-Senftenberg, Cottbus, 03046 Germany

**Keywords:** Engineering, NEMS, Sensors

## Abstract

Electrostatic micromechanical actuators have numerous applications in science and technology. In many applications, they are operated in a narrow frequency range close to resonance and at a drive voltage of low variation. Recently, new applications, such as microelectromechanical systems (MEMS) microspeakers (µSpeakers), have emerged that require operation over a wide frequency and dynamic range. Simulating the dynamic performance under such circumstances is still highly cumbersome. State-of-the-art finite element analysis struggles with pull-in instability and does not deliver the necessary information about unstable equilibrium states accordingly. Convincing lumped-parameter models amenable to direct physical interpretation are missing. This inhibits the indispensable in-depth analysis of the dynamic stability of such systems. In this paper, we take a major step towards mending the situation. By combining the finite element method (FEM) with an arc-length solver, we obtain the full bifurcation diagram for electrostatic actuators based on prismatic Euler-Bernoulli beams. A subsequent modal analysis then shows that within very narrow error margins, it is exclusively the lowest Euler-Bernoulli eigenmode that dominates the beam physics over the entire relevant drive voltage range. An experiment directly recording the deflection profile of a MEMS microbeam is performed and confirms the numerical findings with astonishing precision. This enables modeling the system using a single spatial degree of freedom.

## Introduction

Coulomb-actuated microbeams are beams of microscopic dimensions deformed by the application of electrostatic forces. In addition to membranes, they are among the most important components used for capacitive actuation and sensing in microelectromechanical systems (MEMS)^1–3^. Recent advances in MEMS modeling and design enabled pioneering devices for medical applications (Bio-MEMS)^4^, communication systems (radio-frequency MEMS, RFMEMS)^5^, environmental sensing^2^, and consumer products^6^. Prominent examples are microresonators^7–9^, micropumps^10,11^, accelerometers^12,13^, gyroscopes^14,15^, capacitive micromachined ultrasonic transducers (CMUTs)^16,17^, microphones^18,19^, and microloudspeakers^20,21^. Global trends such as the 5G Internet of Things (5G-IoT)^22^, augmented reality^23^, and Green ICT (information and communications technology)^24,25^ drive a system complexity that can only be handled by introducing lumped-parameter modeling at the MEMS level; an ab initio approach seems impractical. This requires a deep understanding of the electromechanics of the MEMS components involved.

Coulomb-actuated microbeams are commonly modeled as Euler-Bernoulli beams^7,26^ in regard to lumped-parameter modeling. The models of Nayfeh, Younis, and Rahman^27–33^ represent the current state-of-the-art in this field. These authors proposed a modal decomposition technique to analyze the static and dynamic behavior. Unfortunately, Younis et al. encountered convergence issues (for example, reported in Fig. 3 in ref. ^29^). At least three symmetrical modes are needed in their approach to reach decent accuracy. More modes do not necessarily mean more accuracy. Furthermore, all of this also comes at high computational complexity compared to numerical solvers^29^.

**Fig. 1:**
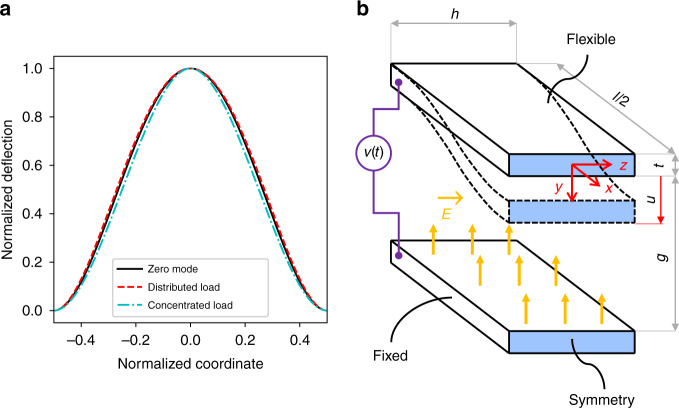
Coulomb-actuated microbeam. **a** The bending profile of a symmetrically loaded Euler-Bernoulli beam: Euler-Bernoulli zero-mode (black solid line), constant distributed load (red dashed line), and fully concentrated load (cyan dash-dotted line). **b** Schematic of a prismatic Coulomb-actuated microbeam in undeflected (solid lines) and deflected (dashed lines) states including characteristic dimensions and the coordinate system.

Classical mechanics suggests to the contrary that there hardly is a need to include higher spatial modes into lumped-parameter models for the type of beams and load situations relevant to MEMS components. The deflection profile of an Euler-Bernoulli beam, clamped at both ends and subject to a symmetric unidirectional load, is almost independent of the spatial distribution of load magnitude^34^. Within very small error margins, it resembles the shape of the lowest eigenfunction (zero mode) of the simple Euler-Bernoulli eigenvalue equation^33,35,36^

1 $$\frac{{{\mathrm{d}}^4}}{{{\mathrm{d}}\xi ^4}}\psi _n\left( \xi \right) = \lambda _n\psi _n\left( \xi \right)$$

2 $$0 \,<\, \lambda _0 \,< \,\lambda_{1} \,<\, \ldots \,< \,\lambda_{n}\, <\, \ldots$$

where *ψ*_*n*_(*ξ*) is the *n*th eigenfunction, *λ*_*n*_ is the *n*th eigenvalue, and *ξ* is the coordinate along the beam. This fact is illustrated in Fig. 1a, comparing the Euler-Bernoulli zero-mode with the bending profiles of the two limiting load cases: a constant distributed load and fully concentrated load (point load). The explanation for this phenomenon is simple as well as far reaching. The bending profile *w*(*ξ*) resulting from a load *q*(*ξ*) is the solution to the equation

3 $$\frac{{{\mathrm{d}}^4}}{{{\mathrm{d}}\xi ^4}}w\left( \xi \right) = q\left( \xi \right),$$

satisfying appropriate boundary conditions^33,34^. Upon decomposition of the load function with respect to a set of normalized Euler-Bernoulli eigenfunctions^33,36^,

4 $$q\left( \xi \right) = \mathop {\sum}\limits_{n = 0}^\infty {\hat q_n\psi _n\left( \xi \right)}$$

we find for the bending profile:

5 $$w\left( \xi \right) = \mathop {\sum}\limits_{n = 0}^\infty {\frac{{\hat q_n}}{{\lambda _n}}\psi _n\left( \xi \right)}\,.$$

In case the load does not change its sign, we always have $\hat q_0 \,\ne\, 0$. The key observation is now that for an Euler-Bernoulli beam, we have largely separated eigenvalues: $\frac{{\lambda _1}}{{\lambda _0}} \approx 29$ and *λ*_*n*_ grow with *n*^4^ (see Supplementary Section 1, Table S1, and Eq. (S3)). Therefore, the Euler-Bernoulli beam acts as a kind of “low-pass filter”, allowing only the first spatial mode, the zero mode, to by far dominate the picture. Note that the eigenvalues are directly related to the elastic energy required to excite the respective eigenmode. The eigenvalue separation therefore also implies a dominant impact of the zero mode on the beam dynamics when kinetic energies involved are not too large.

Does this also hold true for Coulomb-actuated microbeams? We certainly have a symmetric load, pulling in only one direction.

If the zero mode in fact dominates the bending of Coulomb-actuated microbeams to very much the same degree as is the case in pure elastomechanics, this implies major simplifications with respect to efficient modeling of the actuator physics. This would mean, for instance, that only one spatial degree of freedom would be enough to simulate the harmonic distortion of a loudspeaker based on such actuators^21^.

The notion that a single mode of oscillation suffices to describe the physics of a Coulomb-actuated microbeam is not self-evident. This stands in sharp contrast with the findings of Younis et al.^29^. Moreover, the singular nature of the Coulomb force itself nurtures substantial doubt that the lesson taken from elastomechanics outlined above is of any use in the MEMS world. After all, the Coulomb force gives rise to the coexistence of stable and unstable equilibrium states, separated by bifurcation points. Does the saddle-node bifurcation, called pull-in^37,38^ and marking the condition for a potential electromechanical collapse, resemble a kind of elastomechanical buckling? This would destroy all prospects to arrive at a powerful zero-mode-based lumped-parameter model that is amenable to direct physical interpretation. Recently, Spitz et al. observed experimentally that the dynamics of a fairly complex cantilever design can in fact be well described by a lumped-parameter model with only a single degree of freedom^39^. This motivated us to revisit the physics of Coulomb-actuated prismatic microbeams analytically, numerically, and experimentally.

The paper is organized as follows. We devise a finite element method (FEM) strategy that can handle the entire bifurcation diagram, including the pull-in instability and all unstable equilibrium states. Next, our FEM approach is validated by applying it to the reference case presented by Gilbert et al.^40^. We then turn to the main question of how much the Euler-Bernoulli zero-mode actually determines the Coulomb-actuated bending profile compared to the contributions of the three next higher modes. The difference between a pure mechanical load and Coulomb actuation, as well as the impact of the pull-in instability, is investigated by varying the beam thickness. In fact, we find that the physics of a Coulomb-actuated, clamped-clamped prismatic microbeam is by far dominated by a single mode, the Euler-Bernoulli zero-mode. Finally, we compare our numerical findings with those of a microbeam experiment.

## Results

### The Coulomb-actuated prismatic Euler-Bernoulli beam

A prismatic Coulomb-actuated microbeam (see Fig. 1b) can be modeled according to the following equation^32^:

6 $$\frac{{\partial ^2w}}{{\partial \tau ^2}} + c\frac{{\partial w}}{{\partial \tau }} + \frac{{\partial ^4w}}{{\partial \xi ^4}} = \left[ {\alpha _1{\int}_{ - \frac{1}{2}}^{ + \frac{1}{2}} {\left( {\frac{{\partial w}}{{\partial \zeta }}} \right)^2} {\mathrm{d}}\zeta + N} \right]\frac{{\partial ^2w}}{{\partial \xi ^2}} + \alpha _2\frac{{v\left( \tau \right)^2}}{{\left( {1 - w} \right)^2}}$$

where *ξ*, *τ*, *c*, and *w*(*ξ*) are dimensionless quantities, denoting the coordinate along the beam, the time, the damping, and the lateral displacement, respectively. In addition, *α*_1_ and *α*_2_ are geometry-dependent parameters, *N* is a dimensionless external tensile axial force, and *v*(*τ*) is the driving voltage. The dimensionless quantities are defined as follows:

7 $$\xi = \frac{x}{l}$$

where *x* are the coordinates along the beam and *l* is the length of the beam in absolute terms;

8 $$w = \frac{u}{g}$$

*u* being the displacement in the direction of the fixed electrode and *g* the electrode gap at zero deflection in absolute terms;

9 $$\alpha _1 = 6\left( {\frac{g}{t}} \right)^2$$

*t* being the beam thickness in absolute terms; and

10 $$\alpha _2 = \frac{{6{\it{\epsilon }}l^4}}{{Et^3g^3}}$$

where *∈* is the dielectric constant of the gap medium and *E* is Young’s modulus of the beam^32^.

For a clamped-clamped beam, we have the boundary conditions

$$w\left( { - \frac{1}{2},\,\tau } \right) = w\left( { + \frac{1}{2},\,\tau } \right) = 0,$$

11 $$\frac{{\partial w}}{{\partial \xi }}\left( { - \frac{1}{2},\,\tau } \right) = \frac{{\partial w}}{{\partial \xi }}\left( { + \frac{1}{2},\,\tau } \right) = 0.$$

In the following, we consider the static deflection of a beam without the presence of an external axial stress. All time derivatives are set to zero, and the equation reduces to the following nonlinear nonhomogeneous equation of fourth order, containing *γ*, which is a nonlocal functional of the deflection *w*(*ξ*)

12 $$\frac{{{\mathrm{d}}^4w}}{{{\mathrm{d}}\xi ^4}} = \gamma \frac{{{\mathrm{d}}^2w}}{{{\mathrm{d}}\xi ^2}} + \alpha _2\frac{{v^2}}{{\left( {1 - w} \right)^2}},$$

13 $$\gamma = \alpha _1\left[ {{\int}_{ - \frac{1}{2}}^{ + \frac{1}{2}} {\left( {\frac{{{\mathrm{d}}w}}{{{\mathrm{d}}\zeta }}} \right)^2} {\mathrm{d}}\zeta } \right].$$

For small deflections, the effect of the stress stiffening can be neglected, and this equation further simplifies to

14 $$\frac{{{\mathrm{d}}^4w}}{{{\mathrm{d}}\xi ^4}} = \frac{{\alpha _2v^2}}{{\left( {1 - w} \right)^2}}.$$

Note that upon reducing the drive voltage *v* to zero, a Coulomb-actuated beam, originally in a stable state, will return to zero deflection. This means that the effect of the Coulomb force in Eq. (14) approaches that of a constant load of magnitude *α*_2_*v*^2^. For further use, we note here that the same situation occurs with thicker beams beyond the scope of Euler-Bernoulli theory.

### The load at the contact singularity

A little bit less obvious than the constant load situation is the load situation reached when starting from an unstable equilibrium and reducing the drive voltage *v* to zero while keeping forces in equilibrium. The critical bending profile *w*_c_(*ξ*) of the equilibrium state eventually reaches (the upper bifurcation point) and touches the counter electrode at the beam center (contact singularity^29^):

15 $$\mathop {{\lim }}\limits_{v \to 0} w\left( \xi \right) = w_{\mathrm{c}}\left( \xi \right) = \left\{ {\begin{array}{*{20}{l}} { =\!1,\,{\mathrm{if}}\,\xi\, =\, 0} \\ { < 1,\,{\mathrm{if}}\,\xi \,\ne\, 0} \end{array}} \right..$$

This causes a strictly positive difference in the shear forces Δ*S*_0_ at the clamping points

16 $$\Delta S = w^{\left( 3 \right)}\left( { + \frac{1}{2}} \right) - w^{\left( 3 \right)}\left( { - \frac{1}{2}} \right),$$

17 $$\mathop {{\lim }}\limits_{v \to 0} \Delta S = \Delta S_0 \,>\, 0.$$

In fact, the contact singularity is reached when the shear force develops a discontinuity at the beam center. This is shown in Supplementary Section 3. To evaluate the implied load situation, we observe

18 $$\Delta S = {\int}_{ - \frac{1}{2}}^{ + \frac{1}{2}} {w^{\left( 4 \right)}} \left( \xi \right){\mathrm{d}}\xi$$

and find upon using the boundary conditions Eq. (11) and Eq. (16)

19 $${\int}_{ - \frac{1}{2}}^{ + \frac{1}{2}} {\left( {w_{\mathrm{c}}^{\left( 4 \right)}\left( \xi \right) - \gamma w_{\mathrm{c}}^{\left( 2 \right)}\left( \xi \right)} \right)} {\mathrm{d}}\xi = \Delta S_0.$$

On the other hand, Eq. (12) together with Eq. (15) implies

20 $$\mathop {{\lim }}\limits_{v \to 0} \left( {w^{\left( 4 \right)}\left( \xi \right) - \gamma w^{\left( 2 \right)}\left( \xi \right)} \right) = 0,\,{\mathrm{if}}\,\xi\, \ne\, 0.$$

In other words, the nonnegative integrand in Eq. (19) has a nonzero Lebesgue measure and is according to Eq. (20) supported in only a single point *ξ* = 0. This is sufficient to conclude that it exists only in the sense of tempered distributions and that it is proportional to the Dirac delta function^36,41^. Therefore, the mechanical load at the upper bifurcation point is a fully concentrated load,

21 $$w_{\mathrm{c}}^{\left( 4 \right)}\left( \xi \right) - \gamma w_{\mathrm{c}}^{\left( 2 \right)}\left( \xi \right) = \Delta S_0\,\delta \left( \xi \right).$$

The mechanical load experienced by a Coulomb-actuated Euler-Bernoulli beam therefore ranges between a constant load and a concentrated load at low voltages. However, the question remains whether the saddle-node bifurcation at the pull-in voltage changes the picture away from the template of the pure elastomechanic scenario.

### Numerical analysis of the beam used by Gilbert et al.

**Fig. 2:**
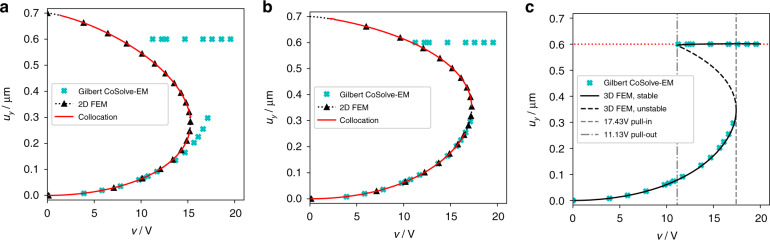
Static deflection curves. **a** Solution without stress stiffening, calculated using two different methods, 2D FEM and the collocation method. Neglecting stress stiffening results in strong disagreement with the results presented by Gilbert et al. **b** Static deflection curves with stress stiffening. The inclusion of stress stiffening leads to good agreement with Gilbert et al. **c** Static deflection curves calculated using 3D FEM including stress stiffening and nonlinear contact. Pull-in and pull-out voltages match the Gilbert et al. results^40^.

To validate our numerical methods, we first reproduced the results of Gilbert et al.^40^. The beam studied by Gilbert et al. had the following geometrical dimensions: beam length *l* = 80 μm, beam width *w* = 10 μm, beam thickness *t* = 0.5 μm, electrostatic gap *g* = 0.7 μm, and stop layer *s* = 0.1 μm. For silicon, Gilbert et al. used an isotropic stiffness with a Young’s modulus of *E* = 169 GPa and a Poisson ratio of *v* = 0.25. The deflection at the center of the microbeam published by Gilbert et al.^40^ is shown in Fig. 2a–c as cyan crosses. This includes two regions: (i) actuation on the stable branch between 0 and 0.3 μm and (ii) maximum displacement, which is constant over the drive voltage above a certain threshold. The latter occurs in the Gilbert case, when the movement of the beam center is stopped at *u*_*y*_ = 0.6 μm by a spacer.

The comparison of the results obtained from applying the collocation solver and our two-dimensional (2D) FEM approach to Gilbert’s geometry, without and with stress stiffening, is displayed in Fig. 2a, b. For the collocation method, the iteration scheme from Eq. (S14) is used; the initial values need to be set carefully to achieve convergence. For the 2D FEM model, the nonlinear geometric effects are activated in ANSYS accordingly. Both methods are capable of solving for unstable states beyond pull-in. It is remarkable that the collocation method can deliver convergent solutions for unstable equilibrium states under all these circumstances. However, the arc-length solver achieves a solution significantly closer to the contact singularity than the collocation solver. Our numerical results are perfectly in line with Gilbert’s findings on the stable branch.

Gilbert’s method generally does not yield points on the unstable branch. The only exception is the leftmost equilibrium state on the upper branch, where a spacer, used in Gilbert’s case, stops the beam. This point lies exactly on the unstable branch of the bifurcation diagram obtained by our FEM approach. Upon lowering the voltage below the threshold corresponding to this contact point, the flexible electrode is released from sticking (pull-out). In principle, spacers can be used to explore more points on the unstable branch.

To analyze the stable contact branch, we extended our 2D FEM model to a three-dimensional (3D) model with nonlinear contact between the microbeam and a spacer, as used by Gilbert. Again, we apply the arc-length solver. The resulting deflection curve is shown in Fig. 2c. Gilbert’s reference curve is now exactly reproduced in full detail. In addition, the complete unstable branch is obtained. This allows us to determine the pull-in and pull-out points much more precisely compared to tracking stable states up to the voltage where a Newton-Raphson solver ceases to converge.

### Modal decomposition as a function of thickness

The relative contributions of Euler-Bernoulli eigenmodes to the deflection profile were computed by means of the FEM and the arc-length method. This calculation was based on Eq. (S10). The impact of the pull-in was studied by a broad variation of the beam thickness of Gilbert’s reference geometry^40^.

**Fig. 3:**
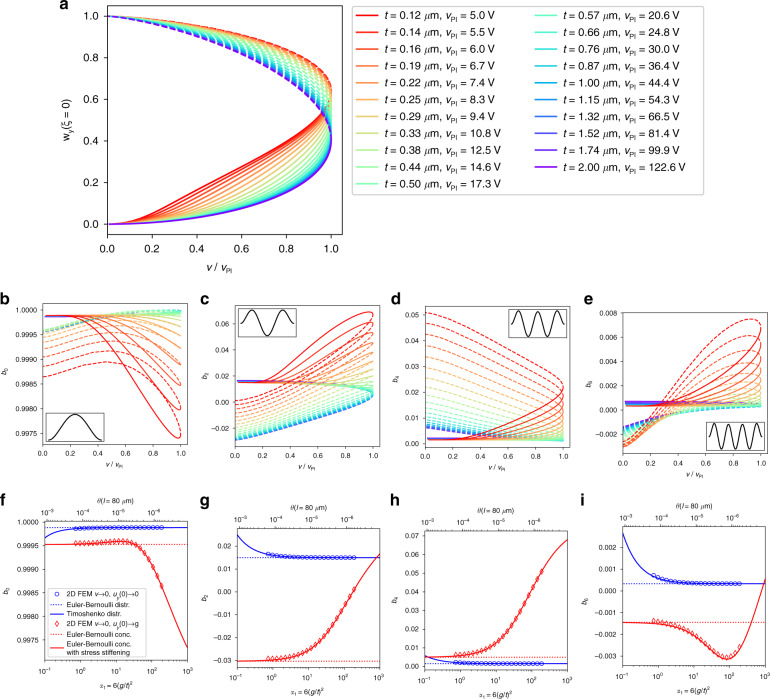
Static deflection and modal contribution. **a** Static voltage–deflection curves of a Coulomb-actuated microbeam for a range of beam thicknesses *t* calculated with 2D FEM. The drive voltage is normalized by the pull-in voltage *v*_PI_, and deflection is normalized by the electrostatic gap *g*. Solid lines indicate the stable branches, and dashed lines indicate the unstable branches. **b–e** Modal contribution extracted from 2D FEM results according to Eq. (10) for the zeroth, second, fourth, and sixth modes, respectively. **f–i** Modal contributions *b*_0_, *b*_2_, *b*_4_, and *b*_6_ for the two limiting cases calculated with the FEM (blue and red markers). The *v* → 0 and *w*(0) → 1 limits (contact singularity) are analytically modeled with Euler-Bernoulli beam theory (red solid line). This limit is the concentrated load case. It is strongly affected by stress stiffening. The limits *v* → 0 and *w*(0) → 0 are analytically modeled with Timoshenko beam theory (blue solid line). This limit is the constant (distributed) load case. In this limit, stress stiffening does not contribute at all. The dotted lines, shown for reference, are the predictions of Euler-Bernoulli beam theory when stress-stiffening and Timoshenko effects are omitted.

The voltage–deflection curves normalized by the pull-in voltage *v*_PI_ and the electrode gap *g* are shown in Fig. 3a for different beam thicknesses *t* in the range between 0.12 and 2 μm. Apparently, the shape of the normalized bifurcation diagram varies significantly with the beam thickness *t*. Thinner beams are shown as orange to red curves. They are more affected by the stress-stiffening effect than thicker beams. Stress stiffening introduces an additional nonlinear restoring force that shifts the pull-in point to higher deflections and changes the shape of the curve. Note that thinner microbeams exhibit an almost linear region between $\frac{v}{{v_{{\mathrm{PI}}}}} = 0.2$ and $\frac{v}{{v_{{\mathrm{PI}}}}} = 0.8$. Such behavior is of interest in many MEMS applications, e.g., in microscale loudspeakers^21,42^.

Only even modes contribute to this symmetric load situation. The relative weights *b*_0_, *b*_2_, *b*_4_, and *b*_6_ of the first four even modes according to Eq. (S10) are shown in Fig. 3b–e as a function of the normalized driving voltage. The Euler-Bernoulli zero-mode has by far the highest relative weight, amounting to at least *b*_0_ ≈ 0.9974. This “worst” case emerges for the thinnest beam (*t* = 0.12 μm) close to the pull-in. The maximum absolute values for the second, fourth, and sixth modes are 0.068, 0.051, and 0.0078, respectively. All these maximums occur for thin beams, while the absolute values significantly drop when *t* increases.

### Comparison of the FEM results with the analytical formula at the contact singularity

Does the FEM solver provide the correct shape of the bending profile after tracking the bifurcation diagram beyond the pull-in point up to the contact singularity? To answer this question, we analytically evaluated Eq. (S10) for the case of a clamped-clamped Euler-Bernoulli beam at the contact singularity as a function of the beam thickness. The result can be found in Supplementary Section 3, Eq. (S24). The picture was completed by adding the constant load case; however, for a Timoshenko beam, see Supplementary Section 4, Eq. (S28). The comparison of the modal contribution calculated analytically with our FEM results is shown in Fig. 3f–i. Note that the graphs for the contact singularity are parametric plots based on Eq. (S22) and Eq. (S24) in Supplementary Section 3, using the stress stiffening *γ* as a parameter.

Figure 3f–i demonstrates a perfect match between the analytical formulae and the 2D FEM results at the contact singularity. The behavior of the beam is governed by the dimensionless quantity *α*_1_ (see Eqs. (6) and (9)). The thinnest FEM-simulated beam has a thickness *t* = 120 nm and a length *l* = 80 μm and behaves closely to a string when stress-stiffening effects are considered. This is reflected in Fig. 3f–i (red line), where the modal contribution of the upper bifurcation point (unstable branch) deviates from linear Euler-Bernoulli theory starting from *α*_1_ = 10^2^. In the case of a lower bifurcation point on the stable branch (Fig. 3f–i, blue line), the modal contribution deviates below *α*_1_ = 10^0^, where a transition to Timoshenko theory occurs. The location of the break-off towards Timoshenko theory is not fixed on the α_1_-axis and is in fact determined by the parameter *θ* in Supplementary Section 3 in Eq. (S25). Although the Euler-Bernoulli assumptions are violated for low and high α_1_ values, we observe in Fig. 3f–i that for both limiting cases in the huge range 10^−1^ < *α*_1_ < 10^3^ (four orders of magnitude), the zero-mode contribution is far above 99%.

### Experimental results

**Fig. 4:**
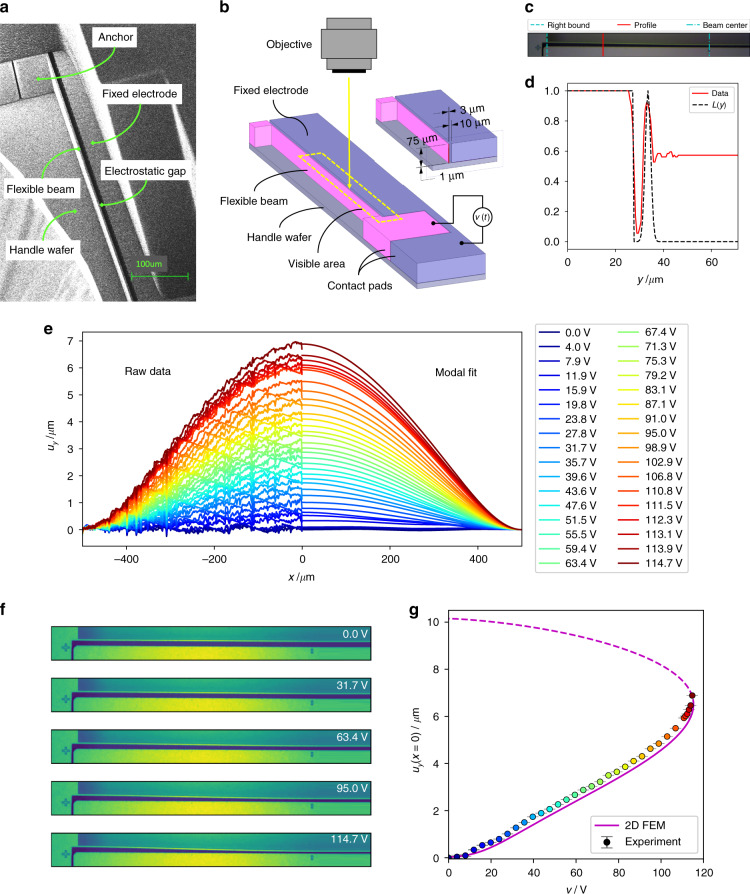
Experimental setup and results. **a** Scanning electron microscope image of the manufactured microbeam. **b** Measurement setup including a perspective view of the full microbeam MEMS device. **c** Microscope image of the microbeam showing lines annotating the beam center (cyan dash-dotted line), the right bound (cyan dashed line), and an exemplary color data profile in-between (red solid line). **d** The normalized color channel data at the exemplary position marked in (**c**). Additionally, shown is the respective fit of the function *L*(*y*) (black dashed line, Eq. (22)). **e** Samples of extracted deflection profiles. The left-hand side shows raw data, and on the right, the data fit to a superposition of the first four symmetric Euler-Bernoulli modes are depicted. **f** Sample beam micrographs at different actuation voltages. **g** Static voltage–deflection curve of the microbeam center. The experimental data are shown as colored circles (colors correspond to the legend in (**e**)), and the FEM results are shown as a magenta line (solid line for the stable branch and dashed line for the unstable branch).

A scanning electron microscope image of the manufactured clamped-clamped microbeam is shown in Fig. 4a, and the optical experimental method for measuring the bending profile is illustrated in Fig. 4b–d. The experimental raw data were fitted to a linear superposition of the first four even Euler-Bernoulli eigenmodes. A subset of the raw data used and the respective fitted bending profiles are shown in Fig. 4e, while a smaller subset of microscopic images is illustrated in Fig. 4f. The coefficients were used to plot the deflection curve (beam center) in Fig. 4g and the modal contributions as functions of the normalized voltage. The fit statistics were used to compute the respective error bars.

**Fig. 5:**
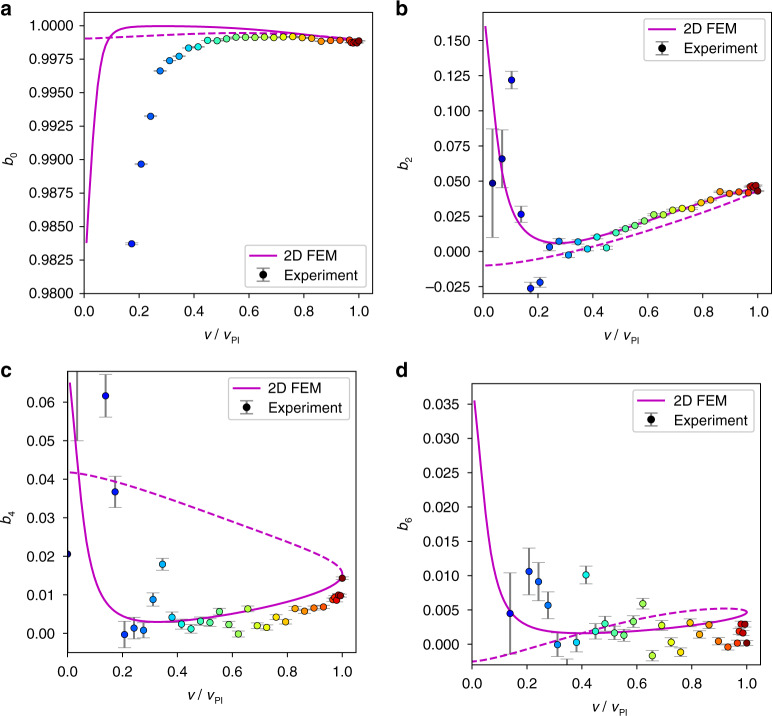
Experimentally determined modal contribution. **a–d** Modal contribution according to Eq. (S10) for the zeroth, second, fourth, and sixth mode. The colored circles show the experimental data and the magenta lines show the FEM results, while the solid line represents the stable branch and the dashed line represents the unstable branch.

Since single-crystal silicon used for manufacturing has unneglectable anisotropy, the 2D FEM model from the previous section was extended by introducing the anisotropic elasticity of crystalline silicon, as provided in the literature (see Eq. (S30) in Supplementary Section 5)^43^. To accurately match the experiment, the manufactured geometry was updated: *t* = 2.47 μm and *g* = 10.15 μm. A small compressive stress of 1.4 MPa was included in the 2D FEM model, which agrees with the experimental observations of Younis et al.^29^. Furthermore, the anchor geometry was included in the model to better approach the real boundary conditions of the microbeam. The modal contribution from experimental data and from FEM simulations (magenta solid and dashed lines) is displayed in Fig. 5. All experimental data are not shown here to avoid unnecessary complexity of the graphs.

**Fig. 6:**
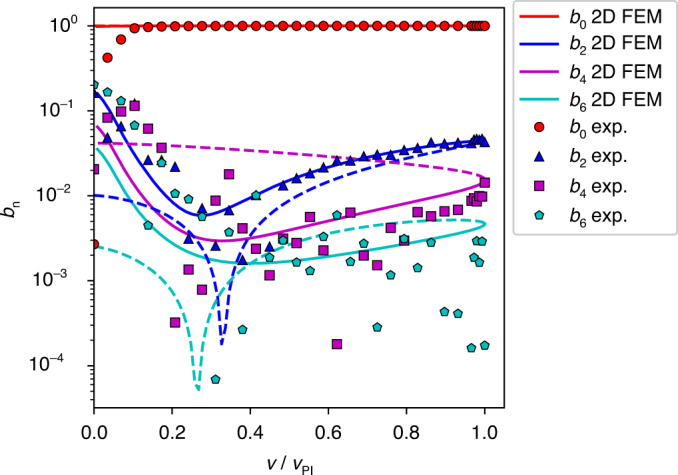
Comparison of modal contributions. Modal contribution of the first four symmetric modes of a silicon microbeam on a logarithmic scale.

The experimental bending profile in Fig. 4g (beam center) and the experimental modal contributions *b*_0_, *b*_2_, and *b*_4_ in Fig. 5a–d are well matched by the FEM simulation. Furthermore, the experiment and 2D FEM simulation are in good agreement regarding a value for the pull-in voltage of approximately *v*_PI_ = 115 V, the pull-in deflection around *u*_*y*_(*x* = 0) = 6.5 μm, and the global shape of the curve (see Fig. 4g). The slight deviations in the deflection could be the result of cumulative effects of local geometric imperfections and 3D-distributed compliance of the anchors, which are attached to the handle on the bottom surface only. Note that the error bars for the deflection curve in Fig. 4g and for *b*_0_ in Fig. 5a are extremely small. The data for *b*_6_ show substantially more scatter (see Fig. 5d), in line with significantly larger error bars. This indicates that tracking more than four modes is beyond the capability of this experiment. According to our FEM analysis, the nature of the anchor design causes small distortions of the Euler-Bernoulli boundary conditions of Eq. (11). Adding geometrical details of the anchor design results in, e.g., the pronounced minimum observed in the *b*_2_ graph (see Fig. 6b). Figure 6 shows a cumulative plot of the considered modal contributions illustrating the tendency of decreasing contribution with increasing *n*. The experiment confirms the dominance of the Euler-Bernoulli zero-mode, despite minor deviations from a pure Euler-Bernoulli situation at the boundary conditions of a real MEMS microbeam.

## Discussion

In Section “Numerical analysis of the beam used by Gilbert et al.”, we demonstrated numerical results for a Coulomb-actuated microbeam with FEM combined with the arc-length method and with the collocation method. Comparison of our analysis with the results of Gilbert et al.^40^ shows perfect agreement, while the FEM in combination with the arc-length solver delivers all bifurcation points and all branches in the 2D and 3D setups. To the best of our knowledge, none of the FEM studies on electrostatically actuated MEMS published so far considers all physically relevant bifurcation points^40,44–51^. The challenge is that at the saddle-node bifurcation (called pull-in instability), the effective stiffness of the system is equal to zero^37,38^, and the widely used Newton-Raphson methods tend to fail accordingly^52,53^. From a computational point of view, this behavior is similar to pure structural instabilities, which can be handled by arc-length solvers^54–56^. We open up this approach here to the evaluation of unstable states of Coulomb-actuated microbeams and other comparable systems. In fact, this method is also applicable to more complex beam designs, which exhibit the coexistence of a multiplicity of stable equilibrium states^42^.

In Section “Modal decomposition as a function of thickness”, we proceeded to apply our FEM-based modal analysis to prismatic beams within a wide thickness range from 0.12 to 2.0 μm. Owing to the accessibility of all bifurcation points, it was possible to numerically observe the modal contribution at the stable and unstable branches, including the saddle-node bifurcation (pull-in) and the state close to contact singularity (*v* → 0 and *u*_*y*_(0) → 1). In Section “Comparison of the FEM results with the analytical formula at the contact singularity”, the existence of analytical solutions for the contact singularity and for the constant distributed load case is highlighted. These analytical solutions confirm the FEM results, and the domination of the zero mode is claimed to hold true for the entire applicable voltage range with a relative zero-mode weight factor of more than 99% (zero-mode hypothesis).

The experimental results reported in Section “Experimental results” substantially differ from the measurements of Coulomb-actuated MEMS presented in the literature^7,8,45,51,57^. The proposed and easily reproducible experimental technique enables accurate static analysis of the beam-bending profile realizable with a common microscope and a voltage supply system. This accuracy allows for the analysis of up to four Euler-Bernoulli modes, which shows excellent agreement with the FEM results on the stable branch. Anisotropic elasticity was considered for comparison of the results; however, in the case of the pure Euler-Bernoulli setting, the elastic properties degenerate to a single elasticity parameter, which is considered in the quantity *α*_2_ and does not change the outcomes. Furthermore, the experimental results confirm that the zero-mode hypothesis holds even in the case of a mild but noticeable violation of the Euler-Bernoulli model assumptions.

**Fig. 7:**
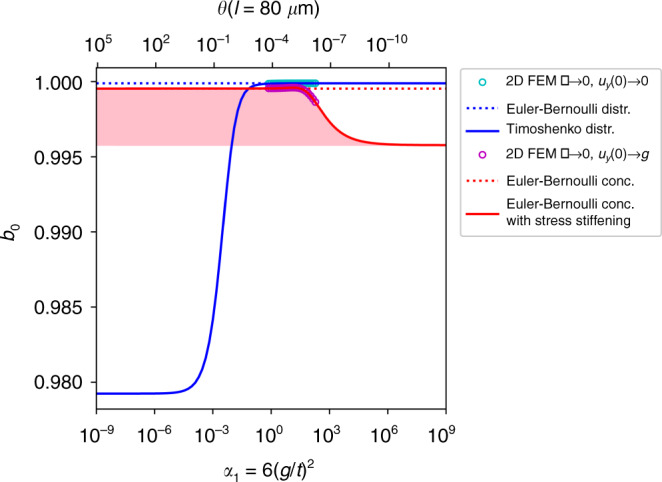
Assessing the boundaries of the Euler-Bernoulli theory. The contact singularity at *v* → 0 and *u*_*y*_(0) → 1 (red solid line) and the limit *v* → 0 and *u*_*y*_(0) → 0 (blue solid line) are analytically calculated as a function of *α*_1_ in an extended range. The Coulomb-Bernoulli zero-mode is a quite accurate approximation to at least approximately *α*_1_ = 10^2^ (see Eq. (9)). At this boundary, the beam begins to develop a string-like behavior. Note that in the string-like region, *b*_0_ is strictly bound by the limits 0.9792 < *b*_0_ < 0.9958 for any value of *α*_1_. The pink area illustrates the range covered by *b*_0_ for *v* > 0. The practical lower bound of the Coulomb-Bernoulli domain is reached at approximately *α*_1_ = 10^−2^ for the beam length selected. More generally speaking, this Timoshenko limit is reached when *θ* attains a value above *θ* = 5 × 10^−3^ (see Supplementary Eq. (S25)). Note that in the Timoshenko region, *b*_0_ is strictly bound by the limits 0.9792 < *b*_0_ < 0.9999 for any value of *θ.*

Figure 7 illustrates the boundaries of the realm of Euler-Bernoulli theory. Reducing the beam thickness eventually affects the transition from a Coulomb-actuated Euler-Bernoulli beam to a Coulomb-actuated string. The Coulomb-actuated string can be analyzed along the lines presented above. This analysis shows that the contact singularity of the Coulomb-actuated string again is the case of a fully concentrated load. The respective bending profile is triangular, as is well known from mechanics^58^. A low electric drive voltage at a low deflection amplitude causes a constant load as the limiting case. The respective bending profile is a parabola^58^. The general normalized bending profiles lie between these two limiting profiles, with the stable profiles being close to the parabola. This allows us to compute the upper and lower bounds of *b*_0_ to be 0.9792 < *b*_0_ < 0.9958 for all values of *α*_1_. The pink area in Fig. 7 lies within the bounds and illustrates the range covered by *b*_0_ when *v* > 0. However, the FEM results in Fig. 3b show that *b*_0_ falls below its value at the contact singularity only above *α*_1_ = 6 × 10^1^ at the verge of applicability of the Euler-Bernoulli theory. The value of the lower bound reflects the fact that the spectral distance of the eigenmodes of a string is much smaller than that of an Euler-Bernoulli beam growing with *n*^2^ rather than with *n*^4^. Therefore, our argument, based on Eq. (5), presented in the introduction does not readily apply to a string. However, the pure string-like case is less of a challenge for modeling since the general bending profiles of the Coulomb-actuated string, including stress stiffening, can be computed analytically. However, it may be of interest in the future to analyze the transitional region more closely.

In the case of thick beams, i.e., in the realm of Timoshenko theory, the smallest value of *b*_0_ (most unfavorable situation) is obtained in the limit of low drive voltages (see Fig. 3b). These cases can be solved analytically and yield polynomials of up to third degree, depending on the analyzed situation. We obtain the *b*_0_ boundaries of 0.9792 < *b*_0_ < 0.9999. Noticeable deviations from the Euler-Bernoulli scenario are observed for *θ* values above *θ* = 5 × 10^−3^. Thick Timoshenko-like beams are less relevant to the design of electrostatic actuators since they require excessive drive voltages to work properly. In summary, the Euler-Bernoulli zero-mode approximation seems useful, beyond the strict limits of the Euler-Bernoulli theory.

The purpose of this paper is to demonstrate that the bending profile of a Coulomb-activated prismatic microbeam clamped at both ends is almost identical to the shape of the first Euler-Bernoulli eigenmode as defined in Eq. (1). To the best of our knowledge, this aspect is not considered in the state-of-the-art modeling of microbeams, as proposed by Nayfeh, Younis, and Rahman^27–33^. The issues encountered by Younis et al.^29^ originate in their treatment of the Coulomb term in Eq. (6). By either multiplying the entire equation with the Coulomb denominator or alternatively by approximating the Coulomb term by a polynomial in *w*(*ξ*) before employing a Galerkin-type procedure, Younis et al.^29^ change the singular nature of the differential equation. A nonperturbative method is required here to resolve the issue. In the sequel to this paper, we plan to propose such an approach based on techniques originating from analytical probability theory.

## Conclusion

Stable and unstable states of prismatic Coulomb-actuated Euler-Bernoulli beams can be successfully simulated by combining the FEM with arc-length solvers. The resulting model predictions can be experimentally verified by combining direct optical observations with modal analysis. Both approaches confirm that the shape of the bending profile of a Coulomb-activated prismatic microbeam, clamped at both ends, is almost identical to the shape of the Euler-Bernoulli zero-mode. This is true for the entire applicable voltage range with a relative zero-mode weight of more than 99%. This observation paves the way for lumped-parameter models of high accuracy that only use a single spatial degree of freedom, amenable to direct physical interpretation.

In a forthcoming paper^59^, we will present a suitable method to carefully treat the Coulomb singularity in Eq. (6), thereby resolving the apparent discrepancy between our findings and the literature mentioned above. The fruits of all this work are an easy-to-handle and surprisingly accurate zero-mode approximation that has numerous applications in the modeling of the physics of electrostatic MEMS actuators.

## Materials and methods

### FEM analysis using an arc-length solver

The deflection solution of the microbeam can be determined either by numerically solving Eq. (12) using, e.g., the collocation method (see Supplementary Section 2), or by utilizing a more general method called FEM. The latter is a common and very versatile approach to model micromechanical systems and avoids the assumptions made to achieve Eq. (12). Commercial FEM codes routinely handle certain nonlinear phenomena such as large deflections and stress stiffening. Typically, these codes efficiently solve the resulting large system of nonlinear equations employing the Newton-Raphson algorithm or one of its modifications. At the pull-in instability, the tangent matrix approaches an infinite slope, and the Newton-Raphson algorithms tend to fail. Therefore, the use of static FEM is usually confined to the analysis of stable equilibrium states, leaving unstable states inaccessible. Even the accurate determination of the pull-in voltage is cumbersome. In practical applications, often the last convergent voltage is accepted as a surrogate for the actual pull-in voltage.

Purely structural (elastomechanic) instabilities, such as buckling or snap-through, share the phenomenon of the tangent matrix approaching an infinite slope. For elastomechanics, Riks^54^ developed in late 1970s a modification of the Newton method allowing for a continuation beyond these instabilities. Crisfield^55^ improved Riks’ method to allow for easy FEM implementation. Today, this method is referred to as the arc-length method. It is a standard method included in commercial codes such as ANSYS and ABAQUS^56^. In this paper, we use the arc-length method to solve a coupled-field problem including electrostatics and elastomechanics. This allows us to compute the entire bifurcation diagram of a Coulomb-actuated prismatic Euler-Bernoulli beam, including all unstable states.

### The experimental setup

To experimentally determine the beam deflection, a Coulomb-actuated MEMS microbeam was manufactured on a bonded silicon on insulator (BSOI) wafer using deep reactive ion etching (Bosch process); see Fig. 4a, b. The 200 mm BSOI wafer consisted of a 75 μm device wafer p-doped with boron at a concentration of 10^18^ cm^−3^, a 1 μm buried oxide layer (SiO_2_), and a 650 μm handle wafer. The prismatic beam design had a beam length of *l* = 1000 μm, a beam width of *w* = 75 μm, and a beam thickness of *t* = 3 μm. The beam is shown in pink in Fig. 4b and was designed for in-plane movement upon activation by a planar counter electrode (violet) placed at a distance of *g* = 10 μm. The beam was clamped in two prismatic anchors with square cross-sections (50 μm × 67.7 μm on the left, 237 μm × 228 μm on the right) and isolated from the rest of the chip by a trench of width 3 μm. To allow for beam movement, the buried oxide layer was removed underneath the beam by means of HF (hydrogen fluoride) vapor etching, and the beam was operated under ambient atmospheric conditions. The actual geometry after fabrication was determined by microscopy, revealing an actual average thickness of *t* = 2.47 μm and an electrostatic gap of *g* = 10.15 μm. To quantify the oxide underetching underneath the anchors of estimated 13 μm, beams were detached from the MEMS chip and inspected from the bottom. A resulting scallop pattern height of approximately 200 nm was determined using scanning electron microscopy. Since the scallop grooves are oriented along the beam axis and their size is small compared to the microbeam dimensions, it was concluded that their impact essentially is a contribution to the average beam thickness *t*, included in the value given above.

The bending profile of the microbeam was recorded using a Leica DM8000 M microscope with an objective 20×/0,4 N PLAN L. All pictures were captured using Bandicam 4.5.6 1647 software in MP4 format at a rate of 30 frames per second. Since the objective could not map the entire beam into one picture frame, only the deflection of one half of the microbeam was recorded (see visible area in Fig. 4b). The location of the beam center was marked on the fixed electrode during manufacturing. A driving voltage sweep from 0 to 120 V was supplied by a Keysight B2902A source unit, set to a slew rate of 1 V s^−1^, small enough to avoid dynamic effects. The driving voltage was applied to the MEMS microbeam contact pads by two Imina MiBot microprobe stations equipped with ST-20-0.5 wolfram needles.

The deflection data were extracted directly from the recorded images, and a typical image is shown in Fig. 4c. A total of 3685 frames were recorded, out of which 34 were used. Per frame, 866 cross-sections were evaluated. At every position *x* along the beam, the normalized color channel data for the respective section across the beam were used to determine the beam deflection at this position. The red line in Fig. 4c marks a typical cross-section used. The corresponding color channel data are shown in Fig. 4d. To extract the geometric positions, the heuristic function *L*(*y*),

22 $$L\left( y \right) = G\left( {y - y_G} \right) + H\left( {y_H - y} \right),$$

was fitted to the color channel data. The coordinate *y* is the coordinate along the beam cross-section. *G*(*y*) is the Gaussian function of width *σ*,

23 $$G\left( y \right) = \frac{1}{{\sigma \sqrt {2\pi } }}e^{ - \frac{1}{2}\left( {\frac{y}{\sigma }} \right)^2}$$

and *H*(*y*) is the Heaviside step function,

24 $$H\left( y \right) = \left\{ {\begin{array}{*{20}{c}} { = \!1,\,{\mathrm{if}}\,y\, > \,0} \\ {=\! 0,\,{\mathrm{if}}\,y \,<\, 0} \end{array}.} \right.$$

The fit parameters were *σ*, the position *y*_*G*_ of the middle of the microbeam along the respective cross-section and the position *y*_*H*_ of the relevant edge of the fixed electrode. The respective fit is also shown in Fig. 4d. This finally leads to the raw data for the experimental deflection profile

25 $$u_y^{{\mathrm{exp}}}\left( x \right) = g - \left( {y_G\left( x \right) - y_H\left( x \right)} \right).$$

## Supplementary information

Supplementary materials.

## Data Availability

The data that support the findings of this study are available from the corresponding author upon reasonable request.
