# Supplementary material for: Coulomb-actuated microbeams revisited: experimental and numerical modal decomposition of the saddle-node bifurcation
Source: Microsyst Nanoeng. 2021 May 28;7:41. doi: 10.1038/s41378-021-00265-y (PMC8433289; doi:10.1038/s41378-021-00265-y)
Supplement: Supplementary file 1 — Supplementary materials. [file 41378_2021_265_MOESM1_ESM.pdf]

Supplementary Materials

Coulomb-Actuated Microbeams Revisited: Experimental and Numerical Modal Decomposition of the Saddle-Node Bifurcation

Anton Melnikov,[1] Hermann A. G. Schenk,[2] Jorge M. Monsalve,[1] Franziska Wall,[1] Michael Stolz,[1,3] Andreas Mrosk,[1] Sergiu Langa,[1] and Bert Kaiser[1]

[1)] *Fraunhofer Institute for Photonic Microsystems IPMS, Dresden, 01109 Germany*

[2)] *Arioso Systems GmbH, Dresden, 01109 Germany*

[3)] *Brandenburg University of Technology Cottbus-Senftenberg, Cottbus, 03046 Germany*

SUPPL. 1. SYMMETRIC EULER-BERNOULLI EIGENMODES

For a prismatic beam, clamped at both ends, the Euler-Bernoulli eigenmodes are defined by the eigenvalue equation Eq. (1) subject to the boundary conditions Eq. (11). The eigensystem for even indices, λ_{2n} and $\psi_{2n}(\xi)$, are given by

$$\psi_{2n}(\xi) = \frac{\cosh(\beta_{2n}\xi)}{\cosh(\frac{\beta_{2n}}{2})} - \frac{\cos(\beta_{2n}\xi)}{\cos(\frac{\beta_{2n}}{2})} \ , \tag{S1}$$

$$\lambda_{2n} = \beta_{2n}^4 \ . \tag{S2}$$

where the β_{2n} are the zeros of

$$0 = \tanh\left(\frac{\beta_{2n}}{2}\right) + \tan\left(\frac{\beta_{2n}}{2}\right) \ , \tag{S3}$$

and can be approximated using

$$\begin{aligned} \beta_{2n} = &\left(2n + \frac{3}{2}\right)\pi + \\ &+ 2\exp\left[-\left(2n + \frac{3}{2}\right)\pi\right] + \\ &+ O\left[\exp\left[-\left(2n + \frac{3}{2}\right)\pi\right]\right]^2 . \end{aligned} \tag{S4}$$

Table S1 illustrates numerically determined β_{2n} from Eq. (S3) in comparison to the approximation using only the first term in Eq. (S4).

TABLE S1. Values of β_{2n}. Comparison between numerically calculated and approximated values for β_{2n}.

$2n$	β_{2n}	$(2n + \frac{3}{2})\pi$
0	4.73004	4.71239
2	10.9956	10.9956
4	17.2788	17.2788
6	23.5619	23.5619

It is readily verified that the function in Eq. (S1) satisfies Eq. (1) and the boundary conditions in Eq. (11). Note that the eigenmodes as defined above are ortho-normal,

$$\int_{-\frac{1}{2}}^{+\frac{1}{2}} \psi_{2n}(\xi)\ \psi_{2m}(\xi)\ d\xi = \begin{cases} 1, & \text{if } n = m \\ 0, & \text{if } n \neq m \ . \end{cases} \tag{S5}$$

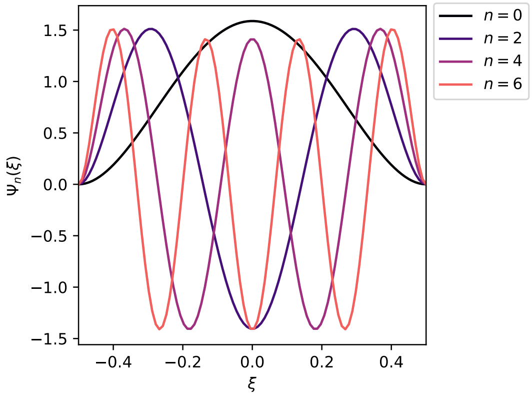

FIG. S1. The first four symmetric normal mode shapes for an Euler-Bernoulli beam in CC condition.

Moreover the even eigenmodes form a complete ortho-nomal base of the Hilbert space of symmetric square integrable functions over the interval $\left[-\frac{1}{2},+\frac{1}{2}\right]$. This means that we can expand any symmetric deflection profile $w(\xi)$ in terms of these eigenmodes

$$w(\xi) = \sum_{n=0}^{\infty} \hat{w}_{2n} \; \psi_{2n}(\xi) \; , \tag{S6}$$

where we have due to ortho-normality

$$\hat{w}_{2n} = \int_{-\frac{1}{2}}^{+\frac{1}{2}} w(\xi) \; \psi_{2n}(\xi) \; d\xi \; . \tag{S7}$$

Note that from these definitions we also get Parseval's equation

$$\int_{-\frac{1}{2}}^{+\frac{1}{2}} w(\xi)^2 \; d\xi = \sum_{n=0}^{\infty} \hat{w}_{2n}^2 \; . \tag{S8}$$

To asses the relative contribution of the individual eigenmodes to the deflection profile we define

$$b_{2n} = \frac{\hat{w}_{2n}}{\sqrt{\sum_{n=0}^{\infty} \hat{w}_{2n}^2}} \; . \tag{S9}$$

Finally, to be able to apply the above formula for the evaluation of the experimental data, we use Parseval's equation

$$b_{2n} = \frac{\int_{-\frac{1}{2}}^{+\frac{1}{2}} w(\xi) \; \psi_{2n}(\xi) \; d\xi}{\sqrt{\int_{-\frac{1}{2}}^{+\frac{1}{2}} w(\xi)^2 \; d\xi}} \; . \tag{S10}$$

SUPPL. 2. THE COLLOCATION METHOD

Eq. (12) can be solved numerically e.g. using a shooting method or a collocation algorithm. We decided to use a fourth order collocation algorithm provided by the SciPy library[1,2], which requires a system of first order differential equations as input. To this end we cast Eq. (12) into a set of four ordinary equations, containing however the integral γ given in Eq. (13)

$$\frac{\mathrm{d}}{\mathrm{d}\xi}\begin{bmatrix} w \\ w_1 \\ w_2 \\ w_3 \end{bmatrix} = \begin{bmatrix} w_1 \\ w_2 \\ w_3 \\ \gamma w_2 + \alpha_2 v^2 (1-w)^{-2} \end{bmatrix}. \tag{S11}$$

In order to deal with γ, we introduce a new function

$$\Gamma(\xi) = \alpha_1 \int_{-\frac{1}{2}}^{\xi} w_1^2 \mathrm{d}\zeta \ , \ \Gamma\left(\frac{1}{2}\right) = \gamma \tag{S12}$$

The function $\Gamma(\xi)$ can be directly obtained during the solution procedure by adding the differential equation

$$\frac{\mathrm{d}}{\mathrm{d}\xi}\Gamma = \alpha_1 w_1^2 \ , \ \Gamma\left(-\frac{1}{2}\right) = 0 \tag{S13}$$

to the system in Eq. (S11). We then define a simple iteration scheme starting with $\gamma_0 = 0$ and solving the new system according to

$$\frac{\mathrm{d}}{\mathrm{d}\xi}\begin{bmatrix} w \\ w_1 \\ w_2 \\ w_3 \\ \Gamma_{i+1} \end{bmatrix} = \begin{bmatrix} w_1 \\ w_2 \\ w_3 \\ \gamma_i w_2 + \alpha_2 v^2 (1-w)^{-2} \\ \alpha_1 w_1^2 \end{bmatrix}, \tag{S14}$$

where i is the iteration index and γ_i is updated using

$$\gamma_{i+1} = \Gamma_{i+1}\left(\frac{1}{2}\right). \tag{S15}$$

The iteration is terminated upon meeting the criterion

$$|\gamma_{i+1} - \gamma_i| = |\Delta\gamma_i| < \epsilon_\gamma, \tag{S16}$$

where ϵ_γ is the targeted accuracy. Depending on parameter settings, a damping constant $c_{\mathrm{ID}} < 1$, adjusting the step size, may be required to reach convergence

$$\gamma_{i+1}^{\mathrm{damped}} = \gamma_i + c_{\mathrm{ID}}\ \Delta\gamma_i. \tag{S17}$$

SUPPL. 3. EULER-BERNOULLI BEAM SUBJECT TO A CONCENTRATED LOAD

In this section we compile the solution to the concentrated load equation (Eq. (21)) including stress-stiffening γ according to Eq. (13) and subject to the boundary conditions Eq. (11). We start by noticing that outside the beam center we need to solve Eq. (20)

$$w_c^{(4)}(\xi) - \gamma\, w_c^{(2)}(\xi) \ = 0 \ \text{ if } \xi \neq 0\ .$$

It is worth emphasising that Eq. (20) holds, irrespective of our claims about the shear force. These can therefore be verified by inspecting the results below. The respective bending profile, compatible with the boundary conditions in Eq. (11) and satisfying Eq. (15) is

$$w_c(\xi) = \begin{cases} c_a\left(\frac{1}{2}-\xi\right) - c_b \sinh\left(\sqrt{\gamma}\left(\frac{1}{4}-\xi\right)\right) - c_c, & \text{if } \xi > 0 \\ c_a\left(\frac{1}{2}+\xi\right) - c_b \sinh\left(\sqrt{\gamma}\left(\frac{1}{4}+\xi\right)\right) - c_c, & \text{if } \xi < 0, \end{cases} \tag{S18}$$

where the the parameters c_a, c_b and c_c are given by

$$\begin{aligned} c_a &= \frac{1}{c_N}\ \sqrt{\gamma}, \\ c_b &= \frac{1}{c_N}\ \operatorname{sech}\left(\frac{\sqrt{\gamma}}{4}\right), \\ c_c &= \frac{1}{c_N}\ \tanh\left(\frac{\sqrt{\gamma}}{4}\right), \\ c_N &= \frac{\sqrt{\gamma}}{2} - 2\ \tanh\left(\frac{\sqrt{\gamma}}{4}\right). \end{aligned} \tag{S19}$$

This may be readily verified by direct computation upon inserting Eq. (S18) into Eq. (20) and into Eq. (15).

The bending profile $w_c(\xi)$, its first and second derivative are continuous at the beam center $\xi = 0$. However the third derivative $w_c^{(3)}(\xi)$ has a discontinuity at this point

$$\begin{aligned} \lim_{\xi\to+0} w^{(3)}(\xi) &= +\frac{1}{2}\Delta S_0 \\ \lim_{\xi\to-0} w^{(3)}(\xi) &= -\frac{1}{2}\Delta S_0 \\ \Delta S_0 &= 2\gamma^{3/2} c_N^{-1} \end{aligned} \tag{S20}$$

This establishes that $w_c(\xi)$ as defined in Eq. (S18) is the bending profile resulting from a concentrated load at the beam center, i.e. it is the solution to Eq. (21). In the limit of a

vanishing stress stiffening we obtain

$$\lim_{\gamma\to 0} \Delta S_0 = 192,$$
$$\lim_{\gamma\to 0} w_c(\xi) = \begin{cases} (1-2\xi)^2(1+4\xi), & \text{if } \xi > 0 \\ (1+2\xi)^2(1-4\xi), & \text{if } \xi < 0\ . \end{cases} \tag{S21}$$

To establish the relation between γ and α_1, required to analytically deal with stress-stiffening at the upper bifurcation point, we use Eq. (13). Performing the necessary integration and solving for α_1 yields

$$\alpha_1 = \frac{\sqrt{\gamma}\left(\sqrt{\gamma} - 4\tanh\left(\frac{\sqrt{\gamma}}{4}\right)\right)^2}{6\sqrt{\gamma} - 2\sqrt{\gamma}\tanh^2\left(\frac{\sqrt{\gamma}}{4}\right) - 24\tanh\left(\frac{\sqrt{\gamma}}{4}\right)}. \tag{S22}$$

For practical applications it is helpful noticing

$$\alpha_1 = \frac{5\gamma}{24} + \frac{\gamma^2}{8064} - \frac{\gamma^3}{2257920} + O\left(\gamma^4\right). \tag{S23}$$

Finally we compute the contributions of the Euler-Bernoulli eigenmodes to $w_c(\xi)$ according to Eq. (S10), which admittedly is a bit tedious

$$b_{2n} = \frac{4\sqrt{3}\gamma^{7/4}}{\beta_{2n}^2\left(\beta_{2n}^4 - \gamma^2\right)}\ \frac{A_{2n}}{\sqrt{B_{2n}}}, \tag{S24}$$

$$\begin{aligned} A_{2n} = {} & -2\gamma + \left(\gamma - \beta_{2n}^2\right)\sec\left(\frac{\beta_{2n}}{2}\right) + \\ & + 2\sqrt{\gamma}\tanh\left(\frac{\sqrt{\gamma}}{4}\right)\beta_{2n}\tanh\left(\frac{\beta_{2n}}{2}\right) + \\ & + \left(\gamma + \beta_{2n}^2\right)\operatorname{sech}\left(\frac{\beta_{2n}}{2}\right), \end{aligned}$$

$$\begin{aligned} B_{2n} = {} & (\gamma - 30)\sqrt{\gamma} + 18\sqrt{\gamma}\tanh^2\left(\frac{\sqrt{\gamma}}{4}\right) - \\ & - 6(\gamma - 20)\tanh\left(\frac{\sqrt{\gamma}}{4}\right). \end{aligned}$$

SUPPL. 4. TIMOSHENKO BEAM SUBJECT TO A CONSTANT LOAD

At very low drive voltages the Coulomb force generates a constant beam load of magnitude $\alpha_1 v^2$. In this section we will compile the relevant formulae. Actually we will do this for the more general case of a Timoshenko beam. This helps us exploring the limits of Euler-Bernoulli theory when varying the beam thickness. In contrast to the Euler-Bernoulli assumptions, Timoshenko beam theory allows for a rotation of the normal to the mid-surface of the beam. The rotation angle is denoted here by $\phi(\xi)$. In the formulae below, $w(\xi)$ is as usual, the displacement in dimensionless form. The Timoshenko beam equations for a prismatic beam of rectangular cross section, subject to a constant unit load then read[3]

$$\begin{aligned} &\frac{\partial^3}{\partial \xi^3}\phi(\xi) = 1, \\ &\frac{\partial}{\partial \xi} w(\xi) - \phi(\xi) + \theta \frac{\partial^2}{\partial \xi^2}\phi(\xi) = 0, \\ &\theta = \left(\frac{12}{10} + \frac{11}{10}\nu_{zx}\right)\left(\frac{g}{l}\right)^2 \frac{1}{\alpha_1}, \end{aligned} \tag{S25}$$

where α_1 is taken from Eq. (9) and the value for ν_{zx} is given in Eq. (S30). The respective boundary conditions are

$$\phi\left(-\frac{1}{2}\right) = \phi\left(+\frac{1}{2}\right) = w\left(-\frac{1}{2}\right) = w\left(+\frac{1}{2}\right) = 0. \tag{S26}$$

The solution to Eq. (S25) is

$$\begin{aligned} \phi(\xi) &= -\frac{1}{6}\xi\left(\frac{1}{4} - \xi^2\right), \\ w(\xi) &= \frac{1}{24}\left(\frac{1}{4} - \xi^2\right)^2 + \frac{1}{2}\theta\left(\frac{1}{4} - \xi^2\right). \end{aligned} \tag{S27}$$

It is readily verified that the function in Eq. (S27) satisfies Eq. (S25) and the boundary conditions in Eq. (S26). Evaluating formula Eq. (S10) using the constant load bending profile yields

$$\begin{aligned} b_{2n} &= \frac{1}{\|w\|}\left(\frac{2}{\beta_{2n}^2}\theta + \frac{4}{\beta_{2n}^5}\tanh\left(\frac{\beta_{2n}}{2}\right)\right), \\ \|w\| &= \left(\frac{1}{362880} + \frac{1}{3360}\theta + \frac{1}{120}\theta^2\right)^{\frac{1}{2}}. \end{aligned} \tag{S28}$$

In case of a pure Euler-Bernoulli beam, i.e. for $\theta = 0$, b_{2n} simplifies to

$$\begin{aligned} b_{2n} &= \frac{1}{\|w\|}\frac{4}{\beta_{2n}^5}\tanh\left(\frac{\beta_{2n}}{2}\right), \\ \|w\| &= \left(72\sqrt{70}\right)^{-1}. \end{aligned} \tag{S29}$$

Note that in the Euler-Bernoulli case, b_{2n} is constant with respect to variations of α_1, i.e it is independent of the thickness of the beam.

SUPPL. 5. ORTHOTROPIC STIFFNESS OF SILICON

The stiffness is applied according to Eq. (8) in Ref.[4] as orthotropic stiffness for [110] crystal direction

$$\begin{aligned} E_x = E_y &= 169\,\mathrm{GPa} \\ E_z &= 130\,\mathrm{GPa} \\ \nu_{yz} &= 0.36 \\ \nu_{zx} &= 0.28 \\ \nu_{xy} &= 0.064 \\ G_{yz} = G_{zx} &= 79.6\,\mathrm{GPa} \\ G_{xy} &= 50.9\,\mathrm{GPa}. \end{aligned} \tag{S30}$$

REFERENCES

[1] P. Virtanen, R. Gommers, T. E. Oliphant, M. Haberland, T. Reddy, D. Cournapeau, E. Burovski, P. Peterson, W. Weckesser, J. Bright, S. J. van der Walt, M. Brett, J. Wilson, K. J. Millman, N. Mayorov, A. R. J. Nelson, E. Jones, R. Kern, E. Larson, C. J. Carey, İ. Polat, Y. Feng, E. W. Moore, J. VanderPlas, D. Laxalde, J. Perktold, R. Cimrman, I. Henriksen, E. A. Quintero, C. R. Harris, A. M. Archibald, A. H. Ribeiro, F. Pedregosa, P. van Mulbregt, and S. . . Contributors, "SciPy 1.0–Fundamental Algorithms for Scientific Computing in Python,".

[2] J. Kierzenka and L. F. Shampine, "A BVP solver based on residual control and the Maltab PSE," ACM Transactions on Mathematical Software (TOMS) **27**, 299–316 (2001).

[3] S. P. Timoshenko and J. N. Goodier, *Theory of elasticity*, 3rd ed., Engineering societies monographs (McGraw-Hill, Auckland, 2004).

[4] M. A. Hopcroft, W. D. Nix, and T. W. Kenny, "What is the Young's Modulus of Silicon?" Journal of Microelectromechanical Systems **19**, 229–238 (2010).
